# Presence of gluten and soy derived excipients in medicinal products and their implications on allergen safety and labeling

**DOI:** 10.1038/s41598-025-95525-6

**Published:** 2025-03-31

**Authors:** Alexandra Figueiredo, Maria Deolinda Auxtero, Adriana Brás, Andreia Casimiro, Isabel Margarida Costa

**Affiliations:** 1 Egas Moniz Center for Interdisciplinary Research (CiiEM), Egas Moniz School of Health & Science, Caparica, 2829-511 Almada, Portugal; 2 Egas Moniz School of Health & Science, Caparica, 2829-511 Almada, Portugal

**Keywords:** Health policy, Public health

## Abstract

Gluten and soy allergies are significant health concerns, particularly in individuals with celiac disease or soy sensitivity. While dietary sources of these allergens are well-studied, their presence in medicinal products remains under-explored. This study assessed the prevalence of gluten and soy-derived excipients in 308 medicinal products authorized for marketing in Portugal. A systematic search of the Summary of Product Characteristics (SmPC) database was conducted for 108 analgesics and antipyretics containing paracetamol, 85 NSAIDs containing ibuprofen, and 115 antiasthmatic and bronchodilator medicinal products. The study found significant associations between pharmacotherapeutic groups and the presence of these allergens (*p* < 0.001). Gluten was more prevalent in the group of analgesics and antipyretics (44.4%) than in NSAIDs (8.2%), whereas soy-derived excipients were more frequent in NSAIDs (14%) than in analgesics and antipyretics (6.5%). No excipients containing gluten or soy were identified in antiasthmatic and bronchodilator medicinal products. In analgesics and antipyretics, 51.2% of solid oral dosage forms and 40% of liquid oral formulations contained gluten. Within the NSAIDs group, gluten was mainly present in liquid oral dosage forms (26.7%). Soy-derived excipients were found in 30% of liquid oral formulations and in 33.3% of rectal dosage forms of analgesics and antipyretics. In the NSAIDs group, soy was more prevalent in liquid oral formulations (26.7%). These findings highlight the need for clearer labeling of allergens in medicinal products and underscore the importance of vigilance for patients with gluten or soy allergies. Further research is required to address gaps in allergen disclosure by pharmaceutical manufacturers and to promote safer medicinal product use for sensitive populations. Enhanced awareness among healthcare providers and patients is essential to mitigate the risk of allergic reactions associated with hidden excipients in medicinal products.

## Introduction

Food allergies are an important public health problem that affects children and adults and their prevalence has increased in the past two to three decades^1^. Gluten, a protein found in wheat, barley and rye can trigger adverse reactions in susceptible individuals.

Gluten ingestion can cause three primary clinical disorders, celiac disease, wheat allergy, and gluten sensitivity, each with distinct characteristics and symptoms^2,3^. Celiac disease (CD) is one of the most prevalent chronic disorders worldwide, with an average prevalence of 1.4% when including individuals diagnosed by serological tests, and an overall prevalence of 0.7% based on biopsy findings^4^. CD causes nutrient deficiency due to impaired absorption of iron, calcium, zinc, vitamin B12, vitamin D, and folate^5^. Wheat allergies are common in children and are triggered by wheat proteins, particularly gluten^6^. Non-celiac gluten sensitivity mimics CD symptoms, but lacks an autoimmune component^7^.

Some excipients are used in various pharmaceutical forms such as starch, pregelatinized starch, sodium starch glycolate, and other excipients derived from wheat, oats, rye, and barley. Corn starch and potato starch may also appear, but these excipients are gluten-free and therefore safe for individuals with gluten-related disorders^8^. Starch, a by-product of gluten extraction, has a wide range of applications in the food and pharmaceutical industries^9^. As a common excipient in medicinal products, starch is the primary source of gluten and can be added to formulations with multiple functions, including gelling, thickening, adhesion, moisture retention, stabilisation, film formation, texturizing, and preventing staling^8,10^.

According to the Food and Agriculture Organization of the United Nations, soy is one of the top eight major food allergens, along with milk, eggs, fish, crustacean shellfish, tree nuts, peanuts and wheat^11^. Soy is considered an allergen by approximately 1.5% of the European population and is a frequent cause of allergies in children^12^. Indeed, soy allergy is the second most common food allergy in childhood (0.4% of children) and at the same time one of the allergies with the highest resolution rate, that is, 25% at the age of four, 45% at the age of six and 69% at the age of ten^13–15^. This allergy depends on the eating habits and the age at which soy is introduced into the diet. Patients with soybean allergies can tolerate other legumes^16^.

Soy contains various proteins with distinct characteristics that pose varying risks for severe allergic reactions. Soy allergies can result in manifestations such as oral allergy syndrome, urticaria, angioedema, rhinoconjunctivitis, asthma, and anaphylaxis^16^. However, according to a study by Savage et al. (2010), the most frequent symptoms after exposure are gastrointestinal symptoms (41%) and skin (28%) symptoms^15^.

Due to its solubility, elasticity, viscosity, adhesiveness, gelling, aggregation, absorption, and emulsification properties^17^, it is found in inhalers, tablets, suppositories, and cosmetic products^18^.

Currently, there is no cure for gluten or soy allergies. The best treatment is complete avoidance of these allergens, not only in diet but also in medicinal products, which requires vigilance in checking product labels for hidden allergen sources. Recent studies indicate that approximately 1.4% of the global population is affected by celiac disease, while soy allergies impact around 1% of Europeans, with higher prevalence observed in children^19^. However, specific prevalence rates for gluten and soy sensitivities in Portugal remain unexplored. To our knowledge, assessment of the presence of gluten and soy excipients in medicinal products is limited.

This study aimed to examine the prevalence of gluten and soy-derived ingredients used as excipients in selected medicinal products authorized for marketing in Portugal, focusing on three commonly used therapeutic classes (analgesics, antipyretics, non-steroidal anti-inflammatory drugs, and antiasthmatics).

## Materials and methods

To identify gluten- and soy-derived excipients in medicinal products, a search was conducted using the Summary of Product Characteristics (SmPC) for human medications authorized for marketing in Portugal. The SmPCs were accessed through the online database INFOMED (https://extranet.infarmed.pt/INFOMED-fo/). The study’s inclusion criteria focused on therapeutic groups among the most prescribed for both children and adults, including analgesics and antipyretics containing paracetamol, non-steroidal anti-inflammatory drugs (NSAIDs) containing ibuprofen.These included analgesics and antipyretics containing paracetamol, non-steroidal anti-inflammatory drugs (NSAIDs) containing ibuprofen, and all antiasthmatics/bronchodilators approved and commercialized in Portugal, regardless of the active ingredient. The antiasthmatics/bronchodilators included in the study represented 19 different active ingredients, with the three most common being fluticasone (29.6%), salmeterol (19.2%), and budesonide (9.9%). According to IQVIA reports, paracetamol accounted for 74.3% of the total sales (in terms of units) within the N02B0 group (non-narcotic analgesics/antipyretics) in Portugal in February 2024, while ibuprofen ranked second, with 8.5% of the total sales. These groups were selected due to their widespread use in addressing common conditions such as pain, fever, inflammation, and respiratory issues, making the findings highly relevant to public health. All medicinal products included in the study required marketing authorization in Portugal, and an associated SmPC available in INFOMED. The study covered both generic and branded medicinal products, in all dosages and formulations, excluding injectables, and included prescription-only as well as over-the-counter medications for pediatric and adult use.

The selection of therapeutic groups also considered their diverse dosage forms (solid, liquid, and rectal), enabling the assessment of allergen presence across various formulations. This comprehensive approach aimed to identify potential risks for sensitive individuals and ensure the findings have practical implications for a broad segment of the population. While the dose or quantity of allergens is important, this information is not provided in the SmPC. Therefore, the study focused solely on identifying the presence of gluten, soy, or similar substances by checking whether any of the excipients listed in Table 1 appeared in the complete list of excipients provided in each SmPC.

**Table 1:** List of excipients that have been researched in the summary of product characteristics (SmPC) and which May contain gluten and soy derivatives.

Gluten derived excipients	Soy derived excipients
Wheat Rye Barley Semolina Bran Malt Glucose syrup Gelatinized starch Pre-gelatinized starch Sodium carboxymethyl starch Modified starch Starch (without indicating the source) Oats Xanthan gum	Soy Lecithin Natural tocopherols Phytosterols and phytosterol esters from soy sources Xanthan gum

Xanthan gum is a polysaccharide derived from the natural fermentation of carbohydrates by *Xanthomonas campestris*. It serves as an effective thickening agent and stabilizer in various applications^20^. Depending on the sources of carbohydrates and proteins used during production (e.g., wheat or soy), xanthan gum can contain traces of gluten or soy proteins. Lecithin is an emulsifier made from soybeans or eggs, which can contain allergenic proteins^21^. Few allergic reactions to soy lecithin have been reported, and the risk of reactions to medicinal products containing soy lecithin is very low. Despite the minimal protein content usually present in soy lecithin, highly sensitive individuals can still react in small amounts. However, due to the presence of case reports, such medicinal products are typically avoided in patients with highly sensitive soy allergies^22^. Although it is unlikely that these products will cause a severe allergic reaction in susceptible individuals and are generally considered gluten-free even when derived from wheat, this possibility cannot be completely ruled out.

Since there is no conclusive proof that both xanthan gum and lecithin compounds are entirely safe for individuals with gluten or soy allergy, this study assumed, as a precautionary measure, that if these excipients were present, the product was not considered allergen-free.

The provided list of excipients was developed based on regulatory documentation, scientific literature, and excipient-specific monographs. All excipients mentioned in the studied SmPCs were reviewed to confirm their inclusion in the list or their classification as allergen-free.

Medicinal products were classified as “non-gluten-free” or “non-soy-free” based on the presence of the components listed in Table 1, as indicated in the SmPC. However, it is important to recognize that cross-contamination during production cannot be completely excluded without explicit confirmation from the manufacturing laboratory. Therefore, the classification in this study relied exclusively on the composition information provided by SmPC.

Statistical analyses were performed with SPSS Statistics version 29.0 for Windows (IBM Corp. Armonk, NY). The level of statistical significance was determined as a two-tailed *p* < 0,05. The chi-squared test and Fisher’s exact test were used to determine significant associations between pharmacotherapeutic groups and the presence of gluten or soy-derived excipients. Frequency was expressed as the percentage of total products containing each excipient.

## Results and discussion

This study analyzed 308 medicinal products, including 108 analgesics and antipyretics containing paracetamol, 85 NSAIDs containing ibuprofen, and 115 antiasthmatic and bronchodilator medicinal products, to determine the presence of gluten and soy-derived excipients. The findings are summarized in Table 2.

**Table 2:** Occurrence of gluten and soy-derived excipients across therapeutic groups and dosage forms.

Dosage form	*N*	Non gluten free	Non soy free
Analgesic and antipyretics with paracetamol	108	48	7
Solid oral dosage forms	86	44 (51.2%)	0
Capsule	4	0	0
Film-coated tablet	18	11 (61.1%)	0
Tablets	50	30 (60%)	0
Prolonged-release tablet	1	1 (100%)	0
Effervescent tablet	8	1 (12.5%)	0
Granules	2	0	0
Effervescent granules	1	0	0
Powder for oral solution in sachet	2	1 (50%)	0
Liquid oral dosage forms	10	4 (40%)	3 (30%)
Syrup	5	3 (60%)	3 (60%)
Oral solution	5	1 (20%)	0
Rectal dosage forms	12	0	4 (33.3%)
Suppository	12	0	4 (33.3%)
NSAIDS with ibuprofen	85	7	12
Solid oral dosage forms	68	3 (4.4%)	8 (11.8%)
Soft capsule	4	0	2 (50%)
Coated tablet	33	1 (3%)	3 (9.1%)
Film-coated tablet	22	2 (9.1%)	3 (13.6%)
Effervescent granules	3	0	0
Granules for oral solution	6	0	0
Liquid oral dosage forms	15	4 (26.7%)	4 (26.7%)
Oral suspension	15	4 (26.7%)	4 (26.7%)
Rectal dosage forms	2	0	0
Suppository	2	0	0
Antiasthmatics and bronchodilators	115	0	0
Inhalation dosage forms	115	0	0
Inhalation powder	61	0	0
Inhalation powder. capsule	14	0	0
Inhalation solution	3	0	0
Solution for inhalation by nebulization	5	0	0
Pressurized solution for inhalation	7	0	0
Suspension for nebulizer inhalation	1	0	0
Pressurized suspension for inhalation	24	0	0

**Fig. 1:**
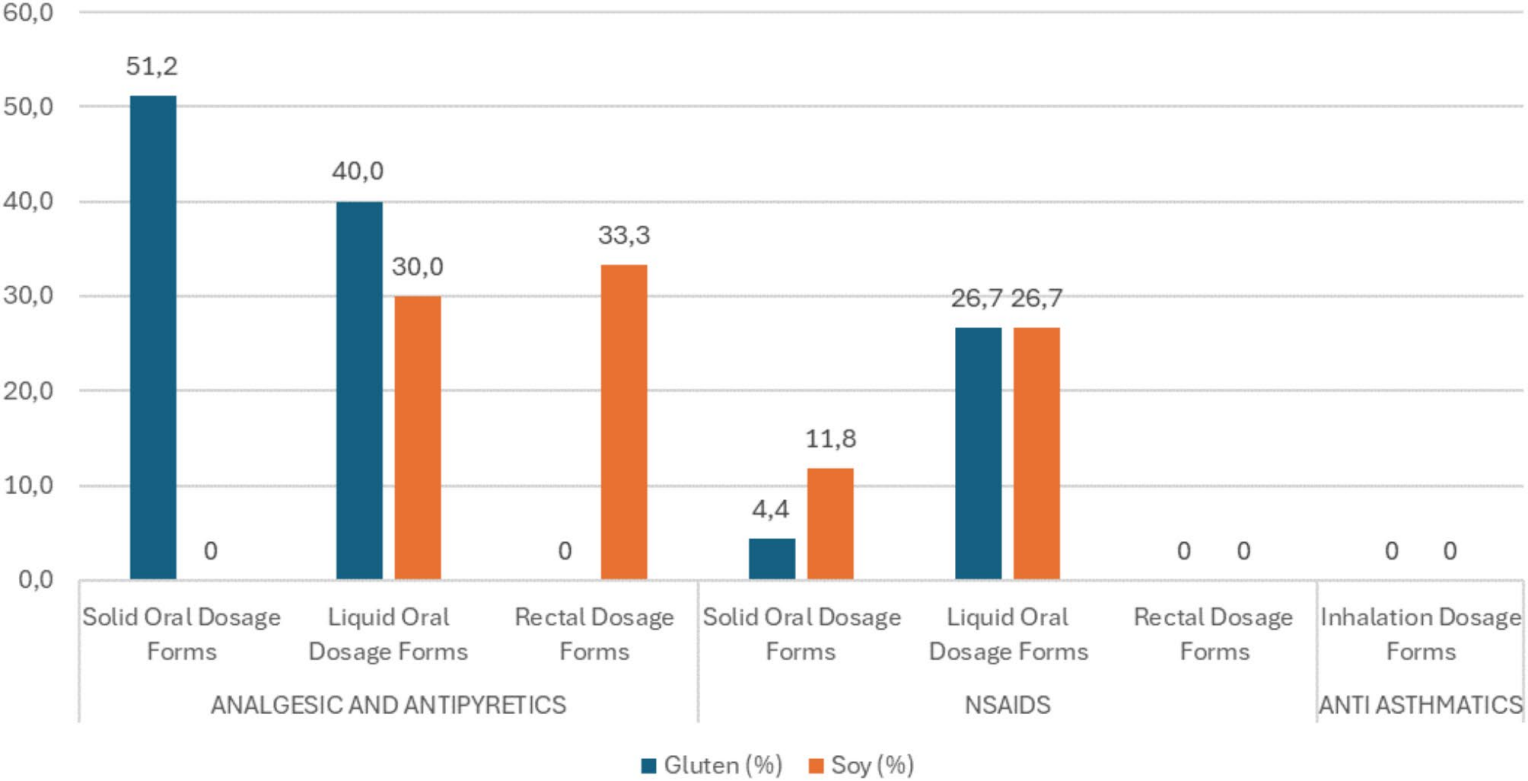
Prevalence of gluten and soy allergens in various dosage forms (%).

The results provide insight into the prevalence of these potential allergens in various dosage forms and their implications for sensitive consumers (Fig. 1).

The presence of individual ingredients from Table 1 was analyzed in Table 3. For gluten-containing excipients, pregelatinized starch and sodium carboxymethyl starch were the most common. The predominant soy-derived excipients were soy and Xanthan gum, which were found exclusively in the analgesic and antipyretic and NSAID groups.

**Table 3:** Gluten and soy excipients listed in the SmPC for each therapeutic group.

	Analgesic and antipyretics^a^	NSAIDs	Antiasthmatics
Excipients which may contain gluten			
Wheat	0	0	0
Rye	0	0	0
Barley	0	0	0
Semolina	0	0	0
Bran	0	0	0
Malt	0	0	0
Glucose syrup	0	0	0
Gelatinized starch	0	0	0
Pre-gelatinized starch	24	0	0
Sodium carboxymethyl starch	22	3	0
Modified starch	2	0	0
Starch	1	0	0
Oats	0	0	0
Xanthan gum	9	4	0
Excipients which may contain soy			
Soy	2	7	0
Lecithin	2	1	0
Natural tocopherols	0	0	0
Phytosterols	0	0	0
Xanthan gum	3	4	0

^a^Ten analgesic and antipyretic products include two excipients that have the potential to contain gluten.

The relationship between pharmacotherapeutic groups and the presence of gluten was analysed using Pearson’s exact chi-squared test, which revealed a strong and significant association (*p* < 0.001). An analogous result (*p* < 0.001) was obtained when the same test was applied to determine the association between the presence of soy and the different pharmacotherapeutic groups. Gluten was more prevalent in the group of analgesics and antipyretics (44.4%) than in NSAIDs (8.2%), whereas soy-derived excipients were more frequent in NSAIDs (14%) than in analgesics and antipyretics (6.5%). No excipients containing gluten or soy were identified in antiasthmatic and bronchodilator medicinal products.

Regarding the prevalence of gluten in medicinal products, this study found that 51.2% of solid oral dosage forms in the analgesic and antipyretic groups were not gluten-free, with film coated tablets (61.1%) and regular tablets (60%) being the most common. In contrast, only 4.4% of the solid oral dose forms in the NSAIDs group contained gluten, indicating a relatively lower prevalence than analgesics and antipyretics. None of the inhalation dosage forms in the anti-asthmatics group contained gluten. The analysis highlighted that solid oral forms, such as tablets and film-coated tablets, in the analgesic and antipyretic groups pose a potential risk to individuals with celiac disease or gluten sensitivity, with gluten present in over 50% of these formulations. However, liquid oral dosage forms and rectal suppositories showed a lower prevalence of gluten, suggesting that these forms might be safer alternatives for gluten-sensitive patients.

Fisher’s exact test was used to assess the association between the presence of gluten and the classification of medicinal products as either generic or branded, irrespective of the therapeutic group. The analysis revealed no significant association (*p* > 0.05) between the presence of gluten and the classification as generic or branded. In contrast, a significant association (*p* < 0.05) was found between the presence of soy and the classification of medicinal products as generic or branded.

Furthermore, the study found that the solid oral dosage forms in the analgesic and antipyretic group did not include soy as an excipient, while 30% of the liquid oral dosage forms contained soy, with syrups (60%) being particularly notable. In the NSAIDs group, 11.8% of the solid oral dosage forms contained soy, with soft capsules (50%) and film coated tablets (13.6%) being the main contributors. The soy content was more prevalent in liquid oral forms (26.7%), presenting a risk to soy allergy individuals.

The presence of gluten- and soy-derived excipients in medicinal products observed in this study is consistent with previous findings that highlight the underestimated risk of allergens in medicinal products. For example, Reker et al. (2019) emphasized that inactive ingredients, including starch derivatives, can exhibit considerable variability between pharmaceutical formulations^23^. Therefore, different commercially available versions of the same medicinal product may contain distinct excipient profiles, potentially posing risks to individuals with heightened sensitivities. Lizano-Díez et al. (2021) highlighted the importance of transparent allergen labeling, noting a lack of standardized declarations for gluten-containing excipients in medicinal products^4^. Similarly, in a survey of the gluten content of 200 drugs, only 18% of manufacturers reported that their drugs contained gluten, while the majority (69%) claimed to produce gluten-free products^24^. However, only 17% tested their products and could provide documentation of the tests performed^24^. Our results further corroborate these observations by demonstrating the variability of allergen presence across drug classes, particularly in oral solid forms of analgesics and NSAIDs.

Despite existing regulations, discrepancies in allergen reporting remain a challenge, particularly for excipients derived from wheat, barley, or soy. While some authors have called for stricter guidelines, others highlight that many medicinal products meet the minimum labeling requirements but fail to address the practical needs of patients managing severe allergies. These gaps underscore the importance of studies like ours to inform regulatory updates and public health initiatives.

These findings have important safety implications for sensitive consumers. Individuals with celiac disease or gluten sensitivity should be cautious when using solid oral dosage forms of analgesics and antipyretics, as these are more likely to contain gluten. Safer alternatives may include liquid oral forms or rectal suppositories. Similarly, individuals with soy allergies should be wary of liquid oral formulations and suppositories across various therapeutic groups, as these have shown a higher prevalence of soy-derived excipients. Despite the low protein content of soy lecithin, highly sensitive individuals should consider avoiding these forms to prevent possible allergic reactions. Healthcare providers should be aware of the potential presence of gluten and soy in medicinal products and advise sensitive patients accordingly. Therefore, they should consider prescribing alternative dosage forms that are less likely to contain these allergens. Manufacturers should clearly label the presence of gluten- and soy-derived excipients to inform healthcare providers and consumers. They may also explore formulations that eliminate these allergens, especially for medicinal products frequently used by sensitive populations. While some companies provide transparency, others may offer incomplete or inaccurate information. Manufacturers often claim not to use gluten but may not guarantee that their products are gluten-free^25^. However, reviewing the list of excipients in a medicinal product provides valuable insight into the potential for gluten or soy contamination.

The legislative framework provides detailed guidance on labeling excipients, yet gaps remain, particularly for derivatives like barley and rye. The EMA’s position on gluten-free thresholds (≤ 20 ppm) is scientifically grounded but requires contextual consideration for highly sensitive populations.

Some SmPCs lacked explicit declarations of excipient origins, highlighting a potential gap in adherence to EMA’s guidelines. These inconsistencies underline the need for harmonization and regular updates. The lack of information on the origin of ingredients used in excipients is a significant concern and a key reason why there is no guarantee regarding the presence of allergens in medicinal products or their risk to allergic patients^8^. When the origin of an excipient is not clearly specified, it may come from various sources, some of which can trigger allergic reactions and compromise patient well-being.

The transparency of allergen data in SmPCs could be improved by leveraging platforms like INFOMED to disseminate excipient origins at minimal cost. Collaboration with patient advocacy groups could complement these efforts to raise awareness and by providing tailored guidance for sensitive populations.

## Strengths and limitations

This study provides novel insights into the prevalence of gluten- and soy-derived excipients in medicinal products marketed in Portugal, focusing on commonly used therapeutic groups. However, its scope is limited to a subset of medicinal products. Additionally, the reliance on SmPCs introduces the possibility of incomplete excipient disclosure. The unavailability of public pharmacovigilance reports also represents a limitation of this study, which restricts the ability to directly correlate allergen presence with adverse reaction reports. The absence of similar studies prevented us from comparing the results of this survey with those of other relevant articles.

## Conclusions

These results highlight the importance of further efforts to minimize allergen exposure in medicinal products and the need for clearer labelling to ensure patient safety. While the overall prevalence of gluten and soy in medications varies between therapeutic groups and dosage forms, the significant associations found in this study emphasize the need for vigilance among sensitive consumers. For individuals with food allergies, determining whether a medicinal product is allergen-free is essential. Proper labeling and increased awareness can help mitigate risks and ensure safer medication use for those with gluten and soy sensitivities.

Current legislation requires manufacturers to disclose the origin of specific excipients, such as wheat starch, when relevant to allergen labeling. However, gaps persist for certain derivatives not explicitly addressed by regulatory frameworks. It is important to recognize that when an excipient is listed as “starch,” it may originate from any starch source, including those that could contain gluten.

This study also underscores the need for more transparent information regarding the origin of excipients in medicinal products. Without such clarity, it is difficult to fully assess the risks for allergic individuals, which remains a concern for both healthcare providers and patients.

## Data Availability

The datasets generated and/or analysed during the current study are not publicly available due to confidentiality concerns regarding the products studied but are available from the corresponding author on reasonable request.
